# Epidermal Growth Factor Receptor (EGFR) Signaling Regulates Global Metabolic Pathways in EGFR-mutated Lung Adenocarcinoma*[Fn FN2]

**DOI:** 10.1074/jbc.M114.575464

**Published:** 2014-06-13

**Authors:** Hideki Makinoshima, Masahiro Takita, Shingo Matsumoto, Atsushi Yagishita, Satoshi Owada, Hiroyasu Esumi, Katsuya Tsuchihara

**Affiliations:** From the ‡ Division of Translational Research, Exploratory Oncology Research & Clinical Trial Center, National Cancer Center, Kashiwa, Chiba 277-8577, Japan,; § Department of Integrated Biosciences, Graduate School of Frontier Sciences, The University of Tokyo, Kashiwa, Chiba 277-8561, Japan,; ¶ Thoracic Oncology Division, National Cancer Center Hospital East, Kashiwa, Chiba 277-8577, Japan, and; ‖ Research Institute for Biomedical Sciences, Tokyo University of Science, Noda, Chiba 278-0022, Japan

**Keywords:** Epidermal Growth Factor Receptor (EGFR), Glucose Metabolism, Glucose Transport, Glycolysis, Lung Cancer, Myc (c-Myc), Pyrimidine

## Abstract

Genetic mutations in tumor cells cause several unique metabolic phenotypes that are critical for cancer cell proliferation. Mutations in the tyrosine kinase epidermal growth factor receptor (EGFR) induce oncogenic addiction in lung adenocarcinoma (LAD). However, the linkage between oncogenic mutated EGFR and cancer cell metabolism has not yet been clearly elucidated. Here we show that EGFR signaling plays an important role in aerobic glycolysis in EGFR-mutated LAD cells. EGFR-tyrosine kinase inhibitors (TKIs) decreased lactate production, glucose consumption, and the glucose-induced extracellular acidification rate (ECAR), indicating that EGFR signaling maintained aerobic glycolysis in LAD cells. Metabolomic analysis revealed that metabolites in the glycolysis, pentose phosphate pathway (PPP), pyrimidine biosynthesis, and redox metabolism were significantly decreased after treatment of LAD cells with EGFR-TKI. On a molecular basis, the glucose transport carried out by glucose transporter 3 (GLUT3) was downregulated in TKI-sensitive LAD cells. Moreover, EGFR signaling activated carbamoyl-phosphate synthetase 2, aspartate transcarbamylase, and dihydroorotase (CAD), which catalyzes the first step in *de novo* pyrimidine synthesis. We conclude that EGFR signaling regulates the global metabolic pathway in EGFR-mutated LAD cells. Our data provide evidence that may link therapeutic response to the regulation of metabolism, which is an attractive target for the development of more effective targeted therapies to treat patients with EGFR-mutated LAD.

## Teaser

**Background:** Genetic mutations in cancer-driver genes induce specific metabolic alterations in cancer cells.

**Results:** EGF receptor signaling has an important role for glycolysis, pentose phosphate pathway, and pyrimidine biosynthesis in EGFR-mutated lung cancer.

**Conclusion:** Our work reveals the relationship between the EGFR signaling axis and key metabolic changes.

**Significance:** These data implicate a possible link between therapeutic response and regulation of metabolism in EGFR-mutated LAD.

## Introduction

The discovery of oncogenic driver mutations allows us to identify druggable targets and develop new therapies using small molecule tyrosine kinase inhibitors (TKIs)^2^ aimed at the relevant patient populations (1–3). More than 50% of lung adenocarcinomas (LAD) from East Asian non-smokers harbor EGFR mutations, and these tumors have been termed oncogene addicted to reflect their dependence on EGFR-mediated pro-survival signaling and their high susceptibility to apoptosis induced by EGFR-TKIs (*e.g.* gefitinib and erlotinib) (4–7). The tyrosine kinase activity of EGFR is dysregulated by gene mutations that lead to aberrant EGFR signaling through pathways including the RAS/MAPK and PI3K/AKT pathways (8, 9). The most frequently occurring mutations in the *EGFR* gene (in-frame deletion in exon 19 at codons 746–750 or a single-base substitution L858R in exon 21) predict an improved clinical response to first-line oral EGFR-TKIs compared with standard platinum-based chemotherapy in patients with advanced non-small-cell lung carcinoma (NSCLC) (4, 8).

There is accumulating evidence that genetic mutations in cancer-driver genes, tumor suppressors, and amplified oncogenes are linked to specific alterations in metabolic activity in cancer cells, involving proteins such as isocitrate dehydrogenase (IDH), fumarate hydratase (FH), MYC, K-RAS, and BRAF (10–13). The Warburg effect, the phenomenon in which cancer cells exhibit rapid glucose consumption with secretion of lactate despite abundant oxygen availability, has been recognized since the 1930s (14–16). Indeed, glucose metabolism in cancer cells is tightly regulated by many molecules at the transcriptional, translational, and post-translational levels (10, 17, 18). c-MYC is critically involved in the regulation of many growth-promoting signal transduction pathways and glucose metabolism genes, including GLUT1, hexokinase 2 (HK2), pyruvate kinase muscle (PKM2), and lactate dehydrogenase A (LDHA) (10, 19). Through the up-regulation of these genes, c-MYC contributes directly to the Warburg effect (19). The enzymatic activities of glycolytic enzymes such as HK2, phosphofructokinase (PFK), PKM2, and LDHA are modulated by post-translational modification (18). For example, PKM2 is phosphorylated in its tyrosine residue (Y105) with low activity in human cancer cells, resulting in increased lactate production, which is one-step downstream from PKM2 in glycolysis, even under aerobic conditions (14, 17). Furthermore, PKM2 promotes the Warburg effect through EGF-stimulated EGFR activation and the MAPK signaling pathway (20, 21). In brain cancer, the activating EGFRvIII mutation induces enhanced glycolysis by promoting glycolytic gene expression through the Myc/Max pathway (22). However, the specific role of mutated EGFR for aerobic glycolysis in lung cancer has not yet been clearly described.

In this work, we demonstrate that EGFR signaling is required for lactate production under aerobic growth conditions in LAD cells. EGFR signaling maintains key metabolites in glycolysis and PPP by regulating glucose transport through GLUT3 expression. In addition to glucose metabolism, we show that EGFR signaling up-regulates *de novo* pyrimidine biosynthesis. Moreover, we describe the altered metabolic profiles in TKI-sensitive LAD cells in response to erlotinib. Our results imply that EGFR signaling plays a central role in modulating global metabolic pathways in EGFR-mutated LAD.

## EXPERIMENTAL PROCEDURES

### 

#### 

##### Materials

Cell lines were purchased from the Immuno-Biological Laboratories (Fujioka, Japan) and American Type Culture Collection (ATCC). RPMI 1640 (R8758 and R1383), phosphate-buffered saline (PBS), 2-deoxy-d-glucose (2DG) were purchased from Sigma-Aldrich. Fetal bovine serum (FBS) was purchased from Biowest (Nuaille, France). Dimethyl sulfoxide (DMSO) and glucose were purchased from Wako Pure Chemicals Industries (Osaka, Japan). Gefitinib and erlotinib were purchased from Santa Cruz Biotechnology (Dallas, TX). Cell Counting Kit-8 was purchased from Dojindo Laboratories (Kumamoto, Japan). Lactate assay kit II and glucose assay kit II were purchased from BioVision (Milpitas). FluxPak XF24 assay pack and XF glycolysis stress test kit were purchased from Seahorse Bioscience (North Billerica). Countess Automated Cell Counter including Trypan Blue and chamber slides was purchased from Invitrogen (Carlsbad, CA). Primary antibodies specific for EGFR, phospho-EGFR Tyr-1068, AKT, phospho-AKT Ser473, ERK1/2, phospho-ERK1/2 Thr202/Tyr204, GSK3α/β, phospho-GSK3α/β Ser21/9, c-MYC, PKM2, phospho-PKM2 Tyr105, GYS, phospho-GYS Ser641, LDHA, phospho-LDHA Tyr-15, HK2, S6K, phospho-S6K Thr421/Ser424, CAD, phospho-CAD (Ser-1859), and β-actin were purchased from Cell Signaling Technologies (Danvers, MA) and GLS, GLUT1, GLUT3, PDHA1, and phospho-PDHA1 Ser-293 from Abcam (Cambridge, UK), respectively. The peroxidase-linked secondary antibodies for WB, HRP-linked Sheep anti-mouse IgG and Donkey anti-rabbit IgG, were purchased from GE Healthcare Biosciences (Pittsburgh, PA). Fluorescein (FITC)-conjugated goat anti-rabbit IgG for FACS was purchased from Beckman Coulter (Fullerton, CA). Oligomycin was purchased from Merck Millipore (Darmstadt, Germany). SYBR Premix Ex Taq was purchased from TaKaRa Bio (Shiga, Japan). Ribonuclease A (RNase A) was purchased from Roche Applied Science (Penzberg, Germany) and contaminated DNase was inactivated at 80 °C for 30 min. 3-*O*-(^3^H-methyl)-*D*-glucose (3-OMeG) was purchased from Perkin Elmer (Waltham, MA).

##### Cell Survival Assay and Proliferation Assay

EGFR mutant LAD cells were seeded in RPMI 1640 containing various concentrations of EGFR inhibitors in 96-well cell culture plates. After 72 h of incubation, cell viability was analyzed using a WST-8 assay using the Cell Counting Kit-8 (Dojindo, Japan). To count the number of viable cells, Trypan Blue-negative cells were counted using a Countess Automated Cell Counter (Invitrogen).

##### Lactate and Glucose Assay

Lactate and glucose in culture medium were measured with the respective lactate assay kit II and glucose assay kit II according to the manufacturer's instructions (BioVision, Mountain View, CA). Briefly, after centrifugation (3,500 rpm, 15 min, 4 °C), cell culture medium supernatants were frozen at −20 °C. Samples were later thawed, diluted in assay buffer, and mixed with lactate or glucose reaction mixture for 30 min. The optical density of the mixture in each well was read at 450 nm on a microplate reader (Molecular Devices). The lactate concentration was calculated from a standard curve and normalized to cell numbers and culture time. Glucose consumption was calculated from a standard curve, subtracting background from cell-free medium, and normalizing to cell numbers and time.

##### Measurement of ECAR and OCR

ECAR and OCR were measured with a XF glycolysis stress test kit according to the manufacturer's instructions (Seahorse Bioscience). In brief, 4.5 × 10^4^ cells were plated onto XF24 plates in RPMI 1640 (10% FBS, 2 mm glutamine) and incubated at 37 °C, 5% CO_2_ overnight. Cells were washed with assay medium (minus glucose and unbuffered RPMI 1640 (SIGMA R1383)), replaced with assay medium, and then placed at 37 °C in a CO_2_-free incubator for 30 min. ECAR and OCR were monitored using a Seahorse Bioscience XF24 Extracellular Flux Analyzer over time and each cycle consisted of 3 min mixing, 3 min waiting and 3 min measuring. Glucose, oligomycin, and 2DG were diluted into XF24 media and loaded into the accompanying cartridge to achieve final concentrations of 10 mm, 5 μm, and 100 mm, respectively. Injections of the drugs into the medium occurred at the time points specified.

##### Western Blotting

Cells were lysed in RIPA buffer (150 mm NaCl, 1% Triton X-100, 0.5% sodium deoxycholate, 0.1% SDS, 50 mm Tris, pH 8.0) on ice for 10 min, sonicated, and centrifuged at 15,000 × *g* for 10 min. The protein content of supernatants was measured by BCA assay (Pierce). Identical amounts of protein samples were separated via 4–20% SDS/PAGE, transferred to PVDF membranes, and incubated overnight with primary antibodies (1:1000 dilution). The primary antibodies used in this study are listed in the materials. ECL anti-rabbit IgG HRP-linked whole antibody (1:10,000; GE Healthcare) and ECL anti-mouse IgG HRP-linked whole antibody (1:10,000; GE Healthcare) were used as secondary antibodies. Signals were detected using ECL Western blotting detection reagent (GE Healthcare) and x-ray films (GE Healthcare).

##### Quantitative RT-PCR

Cells were washed with PBS and total RNA from the LAD cell lines was isolated with TRIzol Reagent (Invitrogen). Complementary DNA (cDNA) was synthesized using the SuperScript VILO cDNA synthesis kit (Invitrogen). Synthesized primers were purchased from TaKaRa Bio (Japan). Real-time RT-PCR was carried out with specific primers and a 7500 detection system (Applied Biosystems). β-Actin was used for normalization as control and the relative quantitation value compared with the calibrator for that target is expressed as 2^−(Ct−Cc)^.

##### Metabolite Measurements

Metabolic extracts were prepared from 2–5 × 10^6^ cells with methanol containing Internal Standard Solution (Human Metabolome Technologies; HMT, Inc., Tsuruoka, Japan) and analyzed using a capillary electrophoresis (CE)-connected ESI-TOFMS and CE-MS/MS system (HMT, CARCINOSCOPE). 2–5 × 10^6^ cells were used for the extraction of intracellular metabolites. Culture medium was removed from the dish, and cells were washed twice in 5% mannitol solution (10 ml first and then 2 ml). Cells were then treated with 800 μl of methanol and 550 μl of Milli-Q water containing internal standards (H3304–1002, HMT, Inc., Tsuruoka, Japan). The metabolite extract was transferred into a microfuge tube and centrifuged at 2,300 × *g* and 4 °C for 5 min. Next, the upper aqueous layer was centrifugally filtered through a Millipore 5-kDa cutoff filter at 9,100 × *g* and 4 °C for 120 min to remove proteins. The filtrate was centrifugally concentrated and re-suspended in 50 μl of Milli-Q water for CE-MS analysis. Cationic compounds were analyzed in the positive mode of CE-TOFMS and anionic compounds were analyzed in the positive and negative modes of CE-MS/MS according to the methods developed by Soga *et al.* (23–25). To obtain peak information including *m*/*z*, migration time (MT), and peak area, detected peaks by CE-TOFMS and CE-MS/MS were extracted using automatic integration software (MasterHands, Keio University, Tsuruoka, Japan and MassHunter Quantitative Analysis B.04.00, Agilent Technologies, Santa Clara, CA, respectively). The peaks were annotated with putative metabolites from the HMT metabolite database based on their MTs in CE and *m*/*z* values determined by TOFMS. The tolerance range for the peak annotation was configured at ±0.5 min for MT and ±10 ppm for *m*/*z*. In addition, concentrations of metabolites were calculated by normalizing the peak area of each metabolite with respect to the area of the internal standard and by using standard curves, which were obtained by three-point calibrations.

##### Expression of Glucose Transporter and Glucose Transport Assay

To detect expression of membrane-bound GLUTs, cells were fixed with 80% ethanol and incubated with anti-GLUT3 antibody (Abcam) and stained with the appropriate FITC-conjugated anti-rabbit IgG antibody (Jackson Immuno Research). Quantification of FITC-fluorescent intensity was performed using a FACSCanto II (BD Biosciences). Procedures for 3-OMeG uptake assay were previously described (26). LAD cells were treated with indicated TKIs for 6 h before glucose transport assay. Uptake was performed from 0.5 min to 10 min and radioactivity in the cells was quantified with Tri-Carb 3110TR low activity liquid scintillation analyzer (PerkinElmer).

##### Statistical Analyses

Unless otherwise indicated, results were reported as the mean ± S.D. Statistical analyses were done by two-tailed Student's *t* test. For metabolomic data analysis we used Welch *t* test and *p* values were indicated as *, <0.05; **, <0.01; and ***, <0.001.

## RESULTS

### 

#### 

##### Lactate Production Was Decreased in TKI-sensitive LAD Cells after EGFR-TKI Treatment

**FIGURE 1.**
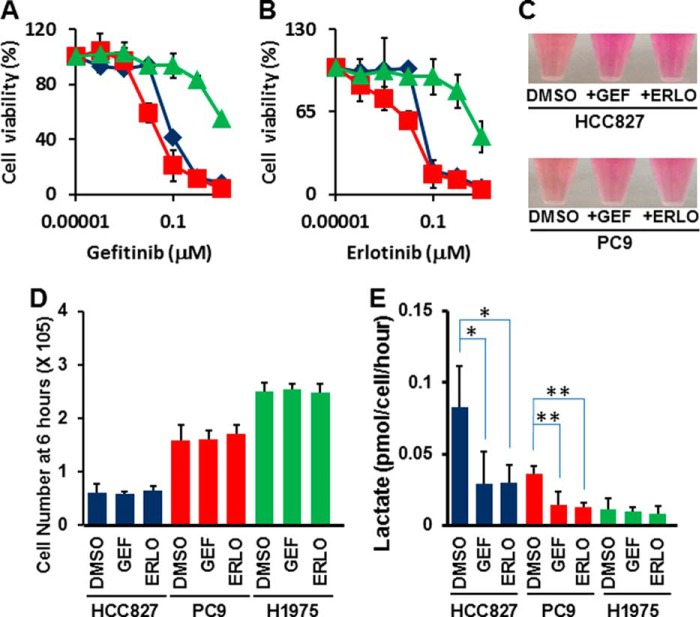
**EGFR-TKI treatment represses lactate production in TKI-sensitive LAD cells.** *A*, WST-8 assay with gefitinib. Cells were treated with the indicated inhibitors for 72 h, and the viability was assessed by the WST-8 assay. Data are shown as the mean ± S.D. (*n* = 6). *Blue line*: HCC827, *red line*: PC-9, *green line*: H1975. The *in vitro* half-maximal inhibitory concentration (IC_50_) for the growth of EGFR-mutated LAD cell lines was determined such that HCC827 to gefitinib: 0.085 μm, PC-9 to gefitinib: 0.031 μm, and H1975 to gefitinib: >10 μm. *B*, WST-8 assay with erlotinib. Cells were treated with the indicated concentrations for 72 h, and viability was assessed by the WST-8 assay. The data are shown as the mean ± S.D. (*n* = 6). *Blue line*: HCC827, *red line*: PC-9, *green line*: H1975. The *in vitro* half-maximal inhibitory concentration (IC_50_) for the growth of EGFR-mutated LAD cell lines was determined such that HCC827 to 0.065 μm, PC-9 to 0.067 μm and H1975 to 8.8 μm. *C*, medium color in HCC827 and PC-9 was altered by addition of EGFR-TKIs (1 μm) for 24 h. The phenol red in culture media exhibits a gradual color transition from red to yellow over the pH range 8.0 to 6.6. *D*, cell growth responses at 6 h to 1 μm of gefitinib or erlotinib were measured using a Trypan Blue staining. The cell number of HCC827 (*blue*), PC-9 (*red*), and H1975 were shown. GEF; gefitinib, ERLO; erlotinib. The data are shown as the mean ± S.D. (*n* = 4). *, *p* < 0.05; **, *p* < 0.01 *versus* control by two-tailed Student's *t* test. *E*, extracellular lactate production in HCC827 (*blue*), PC-9 (*red*) and H1975 (*green*) cell lines at 6 h post-TKI treatment. Error bars indicate S.D. (*n* = 6). *, *p* < 0.05; **, *p* < 0.01 *versus* control by two-tailed Student's *t* test.

We initially characterized the EGFR-mutated lung adenocarcinoma cell lines used in this study by measuring cell viability in the absence or presence of EGFR-TKIs after 72 h. All three LAD cell lines have the EGFR mutation in either exon 19 or exon 21. Cell line HCC827 carried the delE746-A750 mutation, PC-9 exhibited delE746-A750 and NCI-H1975 (H1975) carried EGFR L858R+T790M (27, 28). The H1975 cells have the T790M mutation, which causes resistance to gefitinib and erlotinib (29). HCC827 and PC-9 cell lines were highly sensitive to the EGFR-TKI gefitinib and erlotinib in the nanomolar range as compared with the TKI-resistant cell H1975 (Fig. 1, *A* and *B*). These data are consistent with previous findings (27, 28, 30).

In dose response assays with EGFR inhibitors, we observed differences in the color of the culture medium in the presence of TKIs against EGFR, especially in the growth cultures of TKI-sensitive cell lines (Fig. 1*C*). In culture media, phenol red exhibits a gradual color transition from red to yellow at lower pH values as a result of lactate production (31). Therefore, we explored the glycolytic capacity of EGFR-mutated LAD cells. Since a 72-h incubation with TKIs leads to a dramatic reduction in cell viability in sensitive cell lines, we set up experimental conditions where TKI treatment was given at a relatively higher concentration (1 μm) and shorter time (6 h) to allow all cells to grow equally and thereby standardize the number of viable cells analyzed (Fig. 1*D*). Interestingly, we discovered that exposure of the cells to TKIs for up to 6 h significantly lowered the rate of lactate accumulation in the medium of TKI-sensitive LAD cell lines but not in resistant cells (Fig. 1*E*, *, *p* < 0.05; **, *p* < 0.01 *t* test).

##### Glycolytic Activities Were Down-regulated in TKI-sensitive LAD Cells after Inhibition of EGFR Signaling

**FIGURE 2.**
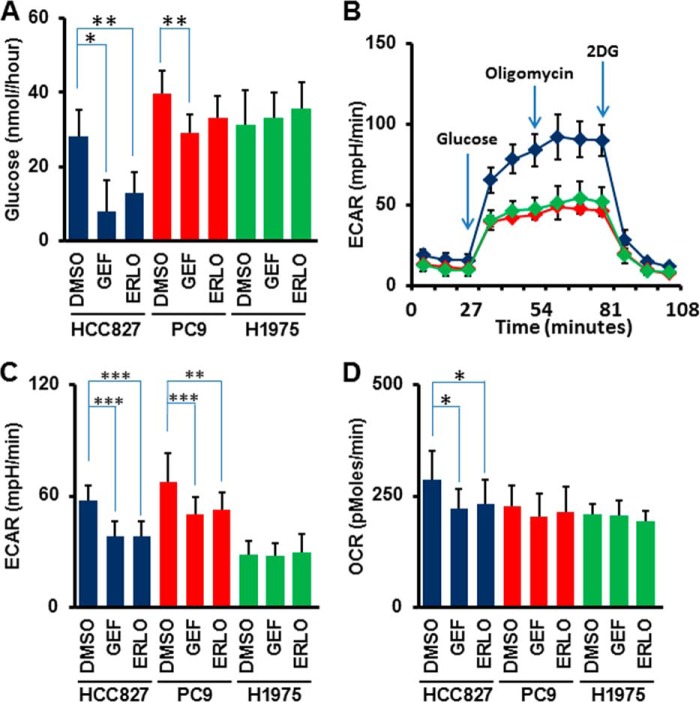
**Glucose consumption and flux analysis monitoring glucose metabolism.** *A*, glucose consumption rate in HCC827 (*blue*), PC-9 (*red*), and H1975 (*green*) cells. Cells were cultured for 24 h in the absence or presence of EGFR-TKIs and glucose concentration in culture supernatant was quantified. Cell-free medium was used as a background control. Error bars indicate S.D. (*n* = 6). *, *p* < 0.05; **, *p* < 0.01 *versus* control by two-tailed Student's *t* test. *B*, measurement of ECAR over time. After 6-h treatment with TKIs, cells were applied to flux assay. ECAR was measured every 9 min. The addition of glucose, oligomycin, and 2-deoxy-d-glucose (*2DG*) was carried out at the time point indicated by the *arrows*. Error bars indicate S.D. *C*, ECAR values of HCC827 (*blue*), PC-9 (*red*), and H1975(*green*) cells at 36 min of flux assay. Error bars indicate S.D. (*n* = 24–30). **, *p* < 0.005; ***, *p* < 0.001 *versus* control by two-tailed Student's *t* test. All cells were treated with the indicated TKIs (1 μm) for 6 h before each assay. *GEF*, gefitinib; *ERLO*, erlotinib. *D*, OCR values of HCC827 (*blue*), PC-9 (*red*), and H1975 (*green*) cells at 36 min of flux assay. Error bars indicate S.D. (*n* = 24–30). *, *p* < 0.001 *versus* control by two-tailed Student's *t* test. All cells were treated with the indicated TKIs (1 μm) for 6 h before each assay. *GEF*, gefitinib; *ERLO*, erlotinib.

Next, we quantified the glucose consumption rate and found that inhibition of EGFR signaling significantly lowered the rate of glucose consumption from the growth medium of TKI-sensitive HCC827 and PC9 cells but not in the TKI-resistant H1975 cells (Fig. 2*A* *, *p* < 0.05; **, *p* < 0.01 *t* test).

To better define lactate production derived from glucose, we measured the glucose-induced extracellular acidification rate (ECAR), an indicator of lactate production, and the oxygen consumption rate (OCR), an indicator of oxidative phosphorylation (OXPHOS), using a flux analyzer. Basal levels of ECAR at the beginning of measurements, which indicated non-glycolytic acidification, were low in HCC827 cells (Fig. 2*B*). Equivalent ECAR was observed in HCC827 cells both pre- and post-treatment with an ATPase inhibitor oligomycin to induce maximum cellular glycolytic capacity (Fig. 2*B*). At the final step, the addition of 2-deoxy-d-glucose (2DG), an inhibitor for glycolysis, completely shut down extracellular acidification (Fig. 2*B*). ECAR was statistically higher in DMSO controls compared with TKI-treated HCC827 and PC-9 cells (Fig. 2*C* *, *p* < 0.01; **, *p* < 0.005; ***, *p* < 0.001 *t* test). In contrast to ECAR, OCR was changed in TKI-treated HCC827, but not in PC-9 and H1975 cells (Fig. 2*D*).

##### EGFR Signaling Up-regulates Glycolysis and the Pentose Phosphate Pathway

**FIGURE 3.**
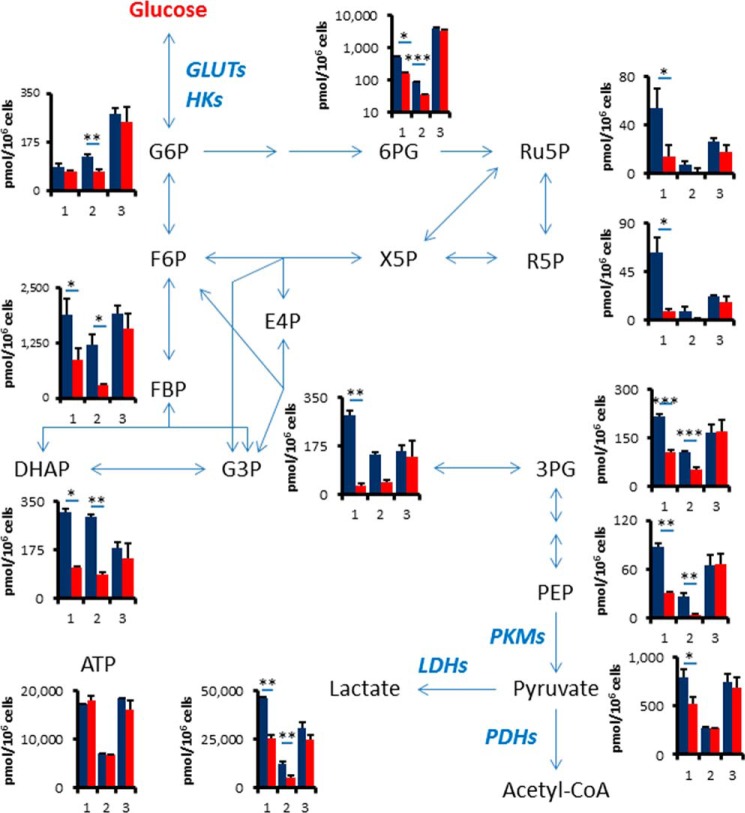
**EGFR signaling up-regulates glycolysis and the pentose phosphate pathway.** Intracellular concentration (pmol/million cells) of key metabolites involved in glycolysis and pentose phosphate pathway (*PPP*) after the inhibition of EGFR signaling is shown. Error bars indicate S.D. (*n* = 3). Total metabolites were extracted with methanol from HCC827, PC9 or H1975 cells treated with DMSO (*blue*) or erlotinib (*red*, 1 μm) for 6 h. Representative metabolites such as glucose 6-phosphate (*G6P*), fructose 1,6-bisphosphate (*FBP*), glyceraldehyde 3-phosphate (*G3P*), dihydroxyacetone phosphate (*DHAP*), 3-phosphoglycerate (*3PG*), phosphoenolpyruvate (*PEP*), pyruvate (*PA*), lactate (*LA*), 6-phosphogluconate (*6PG*), ribulose 5-phosphate (*Ru5P*), ribose 5-phosphate (*R5P*), and ATP are shown here. Others are listed in supplemental Table 1.

To confirm the reduction of glycolysis metabolites by TKIs, we extracted intracellular metabolites with methanol and analyzed using capillary electrophoresis time-of-flight mass spectrometry (CE-TOFMS) (25). Metabolome analysis revealed that intermediate metabolites in glycolysis and the pentose phosphate pathway (PPP) were down-regulated by erlotinib treatment for 6 h in both HCC827 and PC-9 cells (Fig. 3 and supplemental Table S1). We observed that key glycolysis and PPP metabolites such as fructose 1,6-bisphosphate (FBP), dihydroxyacetone phosphate (DHAP), 3-phosphoglycerate (3PG), phosphoenolpyruvate (PEP), lactate (LA), and 6-phosphogluconate (6PG) were decreased in TKI-sensitive HCC827 and PC9 cells after 6 h of erlotinib treatment, but not in TKI-resistant H1975 cells (Fig. 3 and supplemental Table S1). Glucose 6-phosphate (G6P), glyceraldehyde 3-phosphate (G3P), pyruvate (PA), ribulose 5-phosphate (Ribu5P), and ribose 5-phosphate (R5P) were significantly reduced in both HCC827 and PC9 cells. The amount of adenosine triphosphate (ATP), which is the molecular unit of currency of intracellular energy transfer, was not changed in any of the tested three cell lines after erlotinib treatment. The reduction of glucose utilization after TKI treatment was observed in both glycolysis and pentose phosphate pathways, suggesting that EGFR signaling might regulate a glucose transport or hexokinase activity in TKI-sensitive LAD cells.

##### MYC-regulated Gene Expression for Glycolytic Enzymes

**FIGURE 4.**
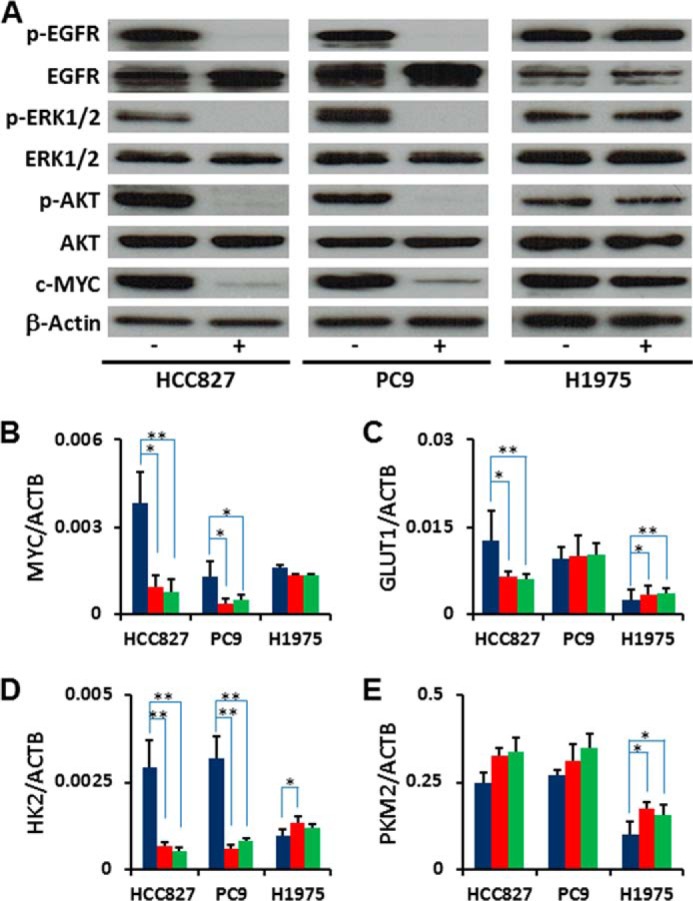
**MYC-regulated glycolytic gene expression.** *A*, Western blotting (*WB*) showing proteins related with EGFR signaling. EGFR, phospho-EGFR (*p-EGFR*), ERK1/2, phospho-ERK1/2 (*p-ERK1/2*), AKT, phospho-AKT (*p-AKT*) and c-MYC. Total protein lysates were isolated from the cell treated with gefitinib for 2 h. Equivalent amounts of proteins from whole-cell lysates were subjected to Western blot analysis to detect the indicated proteins. β-Actin was used as a loading control. *B-E*, total RNA was isolated from cells at 6 h post-TKI treatment and analyzed by RT-PCR. *Blue bars* represent DMSO control, *red bars* denote gefitinib treatment, and *green bars* denote erlotinib treatment. The representative genes related with glycolysis are shown here. *MYC*, *GLUT1* (glucose transporter 1, *SLC2A1*), *HK2* (hexokinase 2), and *PKM2* (pyruvate kinase muscle isozyme 2), and others were listed in supplemental Table S2. Error bars indicate S. D. (*n* = 6). *, *p* < 0.05; **, *p* < 0.01 *versus* control by two-tailed Student's *t* test.

To test whether EGFR-TKIs inhibited EGFR activity and related signaling molecules under our experimental conditions, we determined levels of total EGFR, phospho-EGFR (p-EGFR), ERK1/2, p-ERK1/2, AKT, p-AKT, MYC, and β-actin in cells treated with DMSO or gefitinib (1 μm) for 2 h by Western blot (WB) analyses. Despite equivalent amounts of total EGFR, p-EGFR was clearly repressed in HCC827 and PC9 cells, but not in H1975 cells (Fig. 4*A*). Downstream signaling molecules such as ERK1/2 and AKT were also inactivated by the addition of gefitinib to HCC827 and PC9 cells as compared with H1975 cells (Fig. 4*A*).

We hypothesized that MYC regulates lactate production through transcriptional regulation, since MYC is an important regulator for cell cycle and glycolysis in cancer cells (19, 32, 33). We found that the levels of MYC were quickly down-regulated at both the mRNA and protein levels in response to EGFR-TKIs (Fig. 4, *A* and *B*). The MYC-regulated genes GLUT1 (glucose transporter 1) and HK2 (Hexokinase 2) were down-regulated in EGFR-TKI sensitive LAD cells, but not in the TKI-resistant H1975 cells (Fig. 4, *C* and *D*). Although previous studies reported that MYC up-regulated PKM2 (pyruvate kinase muscle isozyme 2) (34), we found that the loss of MYC did not affect mRNA expression of PKM2 in HCC827 or PC9 cells (Fig. 4*E*). These data suggest that the reduction of glycolysis after the inhibition of EGFR signaling is caused by down-regulation of the MYC pathway.

##### Protein Expression and Phosphorylation of Metabolic Enzymes

**FIGURE 5.**
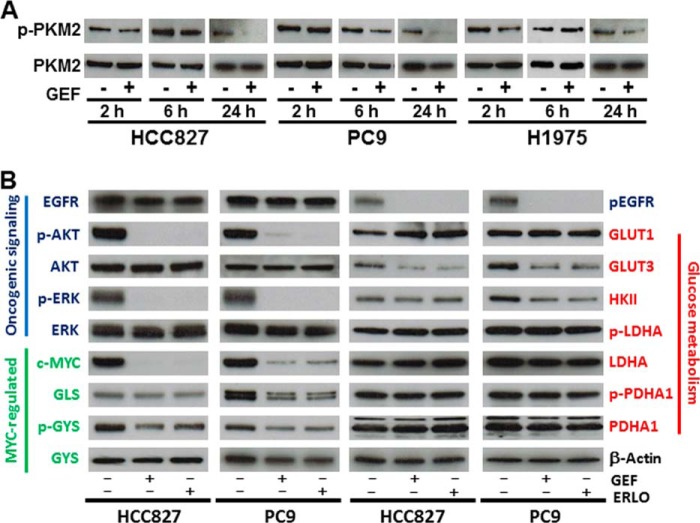
**Protein expression and phosphorylation of metabolic enzymes.** *A*, phosphorylation of PKM2 at Tyr-105 is decreased 24 h after EGFR-TKI addition. EGFR inhibition in the TKI-sensitive HCC827 and PC9 cells did not induce significant reduction of phospho-PKM2 at either 2 or 6 h post-treatment. *B*, Western blotting showing proteins related with oncogenic signaling, GLS, GYS, GLUTs, HKII, PDHA1, and phospho-PDHA1. Total protein lysates were isolated from cells treated with gefitinib or erlotinib for 6 h. Equivalent amounts of proteins from whole-cell lysates were subjected to Western blot analysis to detect the indicated proteins. β-Actin was used as a loading control.

PKM2 is thought to be a key molecule for aerobic glycolysis in cancer cells (20, 21). We hypothesized that PKM2 might play a critical role in the glycolysis pathway in the response to EGFR-TKI treatment, since PKM2 activity was down-regulated by EGF stimulation resulting in up-regulated lactate production (21). Although the phosphorylation of PKM2 at Tyr-105 was decreased at 24 h after EGFR-TKI addition in TKI-sensitive HCC827 and PC9 cells, this was not seen at the earlier time points (2 or 6 h) post-treatment (Fig. 5*A*). The phosphorylation of PKM2 was not changed in TKI-resistant H1975 cells over time (Fig. 5*A*). Therefore, there may be a more rapid, PKM2-independent molecular mechanism by which EGFR signaling regulates the glycolysis pathway.

To determine whether the changes in gene expression driven by MYC were associated with modifications of cellular metabolism, we analyzed phosphorylation and expression of EGFR signaling molecules and glycolytic enzymes by Western blot. We confirmed the effect of TKIs on molecular markers of the EGFR signaling cascade (EGFR, AKT, ERK, and c-Myc) in LAD cells incubated in the presence of gefitinib or erlotinib. We observed that phosphorylation of EGFR, AKT, and ERK was inhibited at 6 h after TKI treatment in HCC827 and PC9 cells (Fig. 5*B*). The expression level of enzymes for glucose metabolism such as GLUT1 and HK2 generally showed regulation in mRNA levels but not at the corresponding protein levels (Fig. 5*B*). In contrast, GLUT3 was decreased in HCC827 and PC-9 cells after 6 h treatment with TKIs (Figs. 4*C* and 5*B*). When we examined the expression level of MYC-regulated glutaminase (GLS) and glycogen synthase (GYS), we saw that GLS and p-GYS were modestly down-regulated in HCC827 and PC9 (Fig. 5*B*). Lactate dehydrogenase A (LDHA), p-LDHA, pyruvate dehydrogenase α1 (PDHA1), and p-PDHA1 were not altered in HCC827 and PC-9 cells after 6 h treatment with TKIs (Fig. 5*B*).

##### EGFR Signaling Pathway Down-regulates the Glucose Transporter GLUT3

**FIGURE 6.**
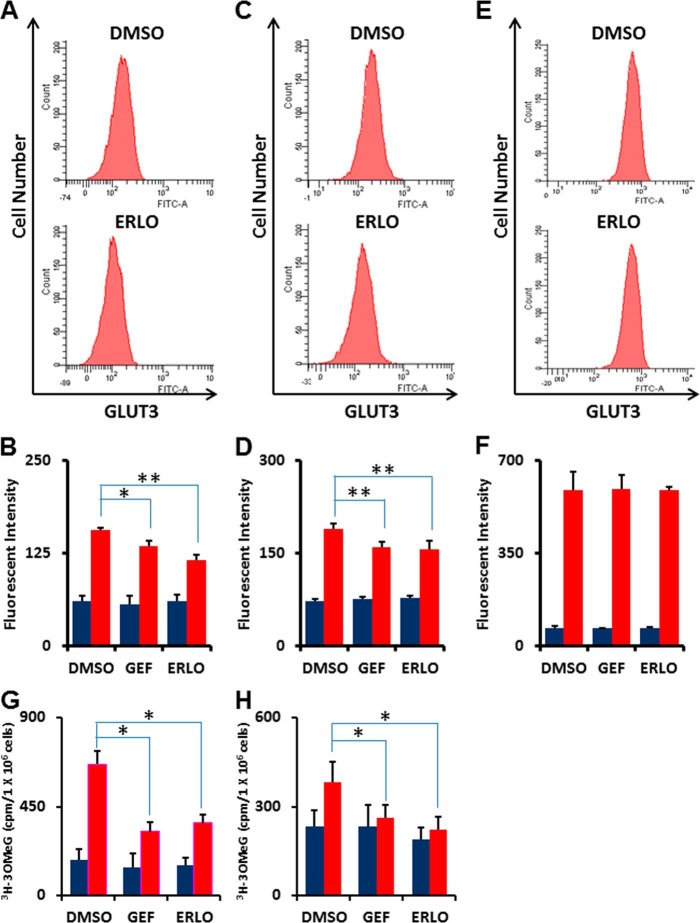
**Glucose transport efficiency is the most rapid and critically regulated function of glucose metabolism linked to EGFR signaling.** *A*, representative flow cytometry plot of GLUT3 expression in HCC827 cells treated with erlotinib (1 μm) or DMSO as a control for 6 h. After fixation, cells were stained with a rabbit anti-GLUT3 antibody and FITC-conjugated anti-rabbit secondary antibody. *B*, flow cytometric analysis of GLUT3 expression in HCC827 cells. *Blue bars* show background fluorescence with IgG isotype control while *red bars* indicate fluorescence staining results with anti-GLUT3 Ab. Error bars indicate S.D. (*n* = 4). *, *p* < 0.05; **, *p* < 0.01 *versus* control by two-tailed Student's *t* test. *C*, representative flow cytometry plot of GLUT3 expression in PC9 cells treated with erlotinib (1 μm) or DMSO as a control for 6 h. After fixation, cells were stained with a rabbit anti-GLUT3 antibody and FITC-conjugated anti-rabbit secondary antibody. *D*, flow cytometric analysis of GLUT1 expression in PC9 cells. *Blue bars* show background fluorescence with IgG isotype control while *red bars* indicate fluorescence staining results with anti-GLUT3 Ab. Error bars indicate S.D. (*n* = 4). *, *p* < 0.05; **, *p* < 0.01 *versus* control by two-tailed Student's *t* test. *E*, representative flow cytometry plot of GLUT3 expression in TKI-resistant H1975 cells treated with erlotinib (1 μm) or DMSO as a control for 6 h. After fixation, cells were stained with a rabbit anti-GLUT3 antibody and FITC-conjugated anti-rabbit secondary antibody. *F*, flow cytometric analysis of GLUT3 expression in TKI-resistant H1975 cells. *Blue bars* show background fluorescence with IgG isotype control while *red bars* indicate fluorescence staining results with anti-GLUT3 Ab. Error bars indicate S.D. (*n* = 4). *, *p* < 0.05; **, *p* < 0.01 *versus* control by two-tailed Student's *t* test. *G* and *H*, 3-*O*-([^3^H]methyl)-d-glucose (3-OMeG) transport efficiency in HCC827 (*G*) and PC-9 (*H*) cells in response to gefitinib or erlotinib as compared with DMSO control. Cells were treated with indicated TKIs (1 μm) for 6 h before transport assay. Radioactivity was measured over time. Cells were harvested at 1 min (*blue*) and 10 min (*red*) after addition of 3-OMeG. Error bars indicate S.D. (*n* = 4). *, *p* < 0.001 *versus* control by two-tailed Student's *t* test.

GLUT1 (glucose transporter 1, *SLC2A1*) and GLUT3 (glucose transporter 3, *SLC2A3*) were mainly expressed in LAD cells (supplemental Table S2). Western blot analysis showed that GLUT3, but not GLUT1, was decreased in HCC827 and PC-9 cells after 6 h of treatment with TKIs. To further confirm membrane-bound GLUT3 expression levels, we investigated the effects of EGFR-TKIs on membrane-bound glucose transporters in HCC827, PC-9, and H1975 cells by flow cytometry. We observed reduction of membrane-bound GLUT3 in the EGFR TKI-sensitive cell lines HCC827 (Fig. 6, *A* and *B*) and PC-9 (Fig. 6, *C* and *D*), although the expression of GLUT3 was unchanged in TKI-resistant H1975 cells (Fig. 6, *E* and *F*) after 6 h of TKI treatment.

To gain additional insight into the functional role of glucose transport, we measured 3-*O*-(^3^H-methyl)-d-glucose (3-OMeG) uptake in the absence or presence of EGFR-TKIs. Following 6-hr treatment with gefitinib or erlotinib, the 3-OMeG transport rate in HCC827 and PC-9 cells significantly decreased (Fig. 6, *G* and *H*).

##### Alterations in Additional Metabolic Pathways other than Glycolysis and PPP in the Response to Erlotinib Treatment

**FIGURE 7.**
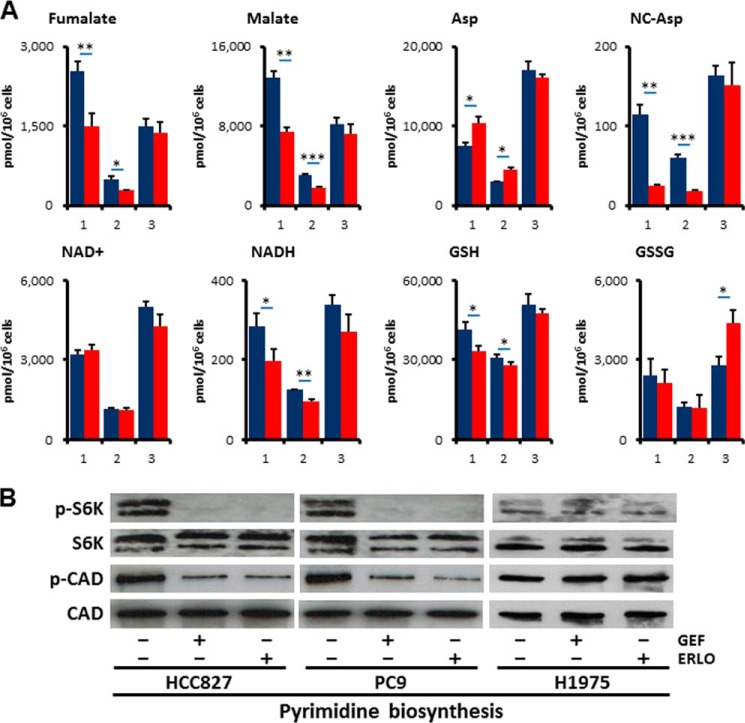
**EGFR signaling maintains *de novo* pyrimidine biosynthesis pathway.** *A*, metabolome analysis. Intracellular concentration (pmol/million cells) of key metabolites involved in glycolysis and pentose phosphate pathway (*PPP*) after inhibition of EGFR signaling is shown. Error bars indicate S.D. (*n* = 3). Total metabolites were extracted with methanol from HCC827, PC9, or H1975 cells treated with DMSO (*blue*) or erlotinib (*red*, 1 μm) for 6 h. Representative metabolites such as fumarate (*FA*), malate (*MA*), aspartate (*Asp*), *N*-carbamoyl-aspartate (NC-Asp), NAD^+^, NADH, reductive glutathione (*GSH*), and oxidative glutathione (*GSSG*) are shown here. *B*, Western blot showing proteins related with *de novo* pyrimidine synthesis. Total protein lysates were isolated from cells treated with gefitinib (1 μm) or erlotinib (1 μm) for 6 h. Equivalent amounts of proteins from whole-cell lysates were subjected to Western blot analysis to detect the indicated proteins. Ribosomal protein S6 kinase 1 (S6K), phospho-S6K (p-S6K, Thr421/Ser424) and carbamoyl-phosphate synthetase 2, aspartate transcarbamoylase, dihydroorotase (CAD), and phospho-CAD (p-CAD, Ser-1859) are shown.

To further characterize whether EGFR signaling regulates additional metabolic pathways other than glycolysis and PPP, we quantified metabolites in tricarboxylic acid (TCA), amino acids, and redox. Despite equivalent amount of acetyl-CoA and citrate, fumarate (FA) and malate (MA) were decreased in HCC827 and PC-9 cells but not in H1975 cells after 6 h treatment with erlotinib (Fig. 7*A* and supplemental Table S1). This result suggests that glutaminolysis was down-regulated after inhibition of EGFR signaling, consistent with the lower expression levels of GLS (Fig. 5*B*). In contrast, amino acids such as proline (Pro) and aspartate (Asp) were increased in TKI-sensitive HCC827 and PC9 cells as compared with TKI-resistant H1975 cells (Fig. 7*A* and supplemental Table S1). Additionally, reductive nicotinamide adenine dinucleotide (NADH) and reductive glutathione (GSH) were significantly reduced in HCC827 and PC9 cells, while conversely oxidative glutathione (GSSG) was increased in H1975 cells (Fig. 7*A* and supplemental Table S1).

##### EGFR Signaling Is Required for de Novo Pyrimidine Biosynthesis

*N*-Carbamoyl-aspartate (NC-Asp) levels were decreased after EGFR-TKI treatment as determined by metabolome analysis (Fig. 7*A*). Consistent with this observation, the phosphorylation of ribosomal protein S6 kinase 1 (S6K) and carbamoyl-phosphate synthetase 2, aspartate transcarbamoylase, dihydroorotase (CAD) were obviously down-regulated in HCC827 and PC9 cells but not in H1975 cells (Fig. 7*B*). The phosphorylation of the Ser1859 residue in CAD protein is required for the first step in the *de novo* synthesis of pyrimidines (35). These data imply that the inhibition of EGFR signaling alters *de novo* pyrimidine biosynthesis in EGFR-mutated LAD cells.

## DISCUSSION

In this report, we demonstrated that EGFR signaling up-regulated aerobic glycolysis in EGFR-mutated LAD cells. EGFR signaling regulates functional GLUT3 to control the glycolysis and pentose phosphate pathways. Moreover, EGFR signaling activated *de novo* pyrimidine synthesis, which is regulated by CAD activity. We conclude that EGFR signaling regulates global metabolic pathways in EGFR-mutated LAD cells. Our data provide evidence that may link the EGFR-TKI response to the regulation of metabolism in EGFR-mutated LAD. Inhibition of EGFR signaling abrogated the Warburg effect by inhibiting multiple steps including MYC-driven transcription and phosphorylation of PKM2 to regulate glycolysis in LAD. We comprehensively quantified the key metabolites in glycolysis and PPP and identified glucose transport as the most important regulatory step for controlling glucose metabolism in LAD cells during EGFR signaling. This observation is consistent with a previous study demonstrating that gefitinib treatment decreased glucose transport efficiency and hexokinase activity in TKI-sensitive LAD cells (26).

The molecular mechanism by which EGFR signaling regulates glucose transport is still unclear. Weihua *et al.* found that EGFR physically associated with and stabilized the sodium/glucose transporter (SGLT1) to promote glucose uptake into cancer cells (36). However, this function did not require EGFR kinase activity. In this report, we found that TKIs to EGFR, gefitinib, and erlotinib, repressed aerobic glycolysis and PPP in EGFR-mutated LAD cells. Although SGLT1 directly interacts with EGFR, EGFR signaling may regulate GLUT translocation in an indirect manner. Mutated EGFRs found in LAD have constitutive tyrosine kinase activity, resulting in activation of downstream RAS/MAPK and PI3K/AKT pathways (Figs. 4*A* and 5*B*). In adipocytes and skeletal muscle, insulin and the PI3K/AKT pathway mediate GLUT4 translocation (37, 38). To promote glucose uptake into muscle and fat cells, insulin stimulates the translocation of GLUT4 from intracellular membranes to the cell surface. Insulin signals go through AS160 (Akt substrate of 160 kDa) and Tbc1Ds to modulate Rab GTPase, and through Rho GTPase TC10a to act on other targets (37, 38). The EGFR-PI3K/AKT axis might control GLUT translocation to the plasma membrane in EGFR-mutated LAD cells. To prove this, we would need to characterize in more detail the molecular mechanisms that control GLUT expression, activity, and translocation.

A recent study showed that AMPK-dependent degradation of thioredoxin-interacting protein (TXNIP) upon stress led to enhanced glucose uptake via GLUT1 (39). Another research report showed that tumor-associated mutant p53 (mutp53) stimulated the Warburg effect in cancer cells as a new mutp53 gain of function (40). Mutp53 did not affect the expression of GLUT1, but promoted aerobic glycolysis by inducing GLUT1 translocation to the plasma membrane, which was mediated by activated RHOA and its downstream effector ROCK. In this study, the EGFR-TKI-sensitive LAD cell lines HCC827 and PC9 possess mutp53, but not the H1975 cell line. A possible molecular mechanism is that either EGFR signaling may regulate GLUT translocation by directly activating the RHOA/ROCK pathway or the mutp53 pathway that in turn activates RHOA/ROCK function. Further experiments would be required to determine whether EGFR signaling controls glucose transport through the TXNIP or mutp53 pathway.

New therapeutic strategies are currently needed to overcome the EGFR T790M-mediated acquired resistance observed in the clinic (8). A recent Phase III study of afatinib monotherapy failed to show overall survival benefit in patients with acquired resistance to reversible EGFR-TKIs(41). Kim *et al.* showed that targeting of glycolysis was an effective therapeutic option to overcome the limited efficacy of afatinib in LAD cells with EGFR T790M (42). Treatment with 2DG completely shut down lactate production in EGFR-mutated LAD cells (Fig. 2*B*), since 2DG is a glucose analog that competes with glucose for cellular uptake. Therefore, combination therapies of EGFR-TKIs and drugs that block the glycolysis pathway such as GLUT-inhibitors would be expected to be much more effective for TKI-resistant cases.

Molecular targeting therapy using TKIs is currently one of the most successful forms of treatment in the clinic, and includes imatinib targeting BCR-ABL in chronic myeloid leukemia (CML) and gefitinib/erlotinib in EGFR-mutated LAD (3). Despite high therapeutic responses to EGFR-TKI treatment, it is clear that not all patients experience benefit; thus, there is still a need to identify potential non-responders and match patients with the most effective therapies (4). Monitoring of tumor glucose utilization by [^18^F]fluorodeoxyglucose (FDG)-positron emission tomography (PET) was implemented for the early prediction of treatment response to EGFR-TKIs in NSCLC (26, 43). In this report, we demonstrate that TKIs to EGFR, gefitinib and erlotinib, repress aerobic glycolysis in EGFR-mutated LAD cells. Those correlations strongly suggest that intermediate metabolites in the pentose phosphate pathway, glycolysis, and pyrimidine biosynthesis such as FBP, DHAP, LA, 6PG, and NC-Asp could serve as well-defined biomarkers to predict response to EGFR-TKI therapy.

The application of metabolomics in oncology has focused its ability to identify biomarkers for cancer diagnosis, prognosis, and therapeutic efficacy (44). In our previous study, we compared the metabolomics of normal and tumor tissues surgically resected pairwise from nine lung patients using CE-TOFMS to elucidate tumor-specific metabolism (45). Significantly high lactate concentrations and elevated activating phosphorylation levels of phosphofructokinase and pyruvate kinase in lung tumors confirmed hyperactive glycolysis (45). Here we show that EGFR signaling regulates many metabolites in EGFR-mutated LAD cells under *in vitro* culture conditions; however, whether EGFR-TKIs have the same effects *in vivo* is still unknown. To build upon this work, further investigations will explore these concepts in relevant animal models and in LAD tissue biopsy samples using bronchoscope before and after EGFR-TKI therapy. *In vivo* validation of these concepts will have significant implications for future diagnostic and therapeutic possibilities for patients.

## Footnotes

2 The abbreviations used are:

TKI: tyrosine kinase inhibitor
EGFR: epidermal growth factor receptor
LAD: lung adenocarcinoma
IC_50_: half maximal inhibitory concentration
PPP: pentose phosphate pathway
ECAR: extracellular acidification rate
OCR: oxygen consumption rate
GLUT: glucose transporter
CAD: carbamoyl-phosphate synthetase 2, aspartate transcarbamylase, and dihydroorotase.
